# Socioeconomic differences in healthy and disease-free life expectancy between ages 50 and 75: a multi-cohort study

**DOI:** 10.1093/eurpub/cky215

**Published:** 2018-10-10

**Authors:** Jenny Head, Holendro Singh Chungkham, Martin Hyde, Paola Zaninotto, Kristina Alexanderson, Sari Stenholm, Paula Salo, Mika Kivimäki, Marcel Goldberg, Marie Zins, Jussi Vahtera, Hugo Westerlund

**Affiliations:** 1 Department of Epidemiology and Public Health, University College London, London, UK; 2 North-East Centre, Indian Statistical Institute, Tezpur, India; 3 Centre for Innovative Ageing, University of Swansea, Swansea, UK; 4 Division of Insurance Medicine, Department of Clinical Neuroscience, Karolinska Institutet, Stockholm, Sweden; 5 Department of Public Health, University of Turku and University Hospital Turku, Turku, Finland; 6 School of Health Sciences, University of Tampere, Tampere, Finland; 7 Department of Psychology, University of Turku, Turku, Finland; 8 Finnish Institute of Occupational Health, Turku, Finland; 9 Clinicum, Faculty of Medicine, University of Helsinki, Helsinki, Finland; 10 Inserm, Population-based Cohorts Unit-UMS 011, Villejuif, France; 11 Stress Research Institute, Stockholm University, Stockholm, Sweden

## Abstract

**Background:**

There are striking socioeconomic differences in life expectancy, but less is known about inequalities in healthy life expectancy and disease-free life expectancy. We estimated socioeconomic differences in health expectancies in four studies in England, Finland, France and Sweden.

**Methods:**

We estimated socioeconomic differences in health expectancies using data drawn from repeated waves of the four cohorts for two indicators: (i) self-rated health and (ii) chronic diseases (cardiovascular, cancer, respiratory and diabetes). Socioeconomic position was measured by occupational position. Multistate life table models were used to estimate healthy and chronic disease-free life expectancy from ages 50 to 75.

**Results:**

In all cohorts, we found inequalities in healthy life expectancy according to socioeconomic position. In England, both women and men in the higher positions could expect 82–83% of their life between ages 50 and 75 to be in good health compared to 68% for those in lower positions. The figures were 75% compared to 47–50% for Finland; 85–87% compared to 77–79% for France and 80–83% compared to 72–75% for Sweden. Those in higher occupational positions could expect more years in good health (2.1–6.8 years) and without chronic diseases (0.5–2.3 years) from ages 50 to 75.

**Conclusion:**

There are inequalities in healthy life expectancy between ages 50 and 75 according to occupational position. These results suggest that reducing socioeconomic inequalities would make an important contribution to extending healthy life expectancy and disease-free life expectancy.

## Introduction

In Europe, life expectancy continues to rise^1^ and disability rates in later life have been falling in Europe and the USA since the 1980s.^2^^,^^3^ However, there are concerns that increases in healthy, disease-free years of life (health expectancies), are no longer keeping pace with those in life expectancy, leading to an expansion of time with morbidity.^4^^,^^5^ Furthermore, there are striking socioeconomic differences in life expectancy, but the extent to which these also exist in health expectancies remains unclear.^1^

The few studies that have examined socioeconomic inequalities in health expectancies found that those in lower socioeconomic groups are doubly disadvantaged by shorter life expectancy and more years spent in ill health.^6^^,^^7^ Results showed that people with higher education not only live longer but spend more years in better health than those with a lower education.^8–12^ Even fewer studies have investigated occupational socioeconomic position, a more proximal measure which may better reflect adulthood circumstances,^13^ and these also suggest that there are socioeconomic differences in health expectancies.^14^^,^^15^ Given that many governments expect people to extend their working lives and that good health is associated with extended working, it is important to study socioeconomic inequalities in health expectancies. In this context, occupational position may be a more relevant indicator than education.

The aim of this multi-cohort study is to contribute to knowledge on inequalities in health expectancies at older ages. We examined occupational class differences in both healthy life expectancy and chronic disease-free life expectancy between the ages of 50 and 75 years for men and women from four cohorts with longitudinal data on health spanning several years.

## Methods

The data came from four cohort studies in England, Finland, France and Sweden. People were included from the first observation with valid data on self-rated health and chronic diseases when they were aged 50 years or older.^16^ We limited our estimation of partial life expectancy to an upper age of 75 as not all cohorts had participants aged 75 and older.

### Samples

The English data were from the first six waves of the English Longitudinal Study of Ageing (ELSA), an open-access, nationally representative biennial longitudinal survey of those aged 50 and over living in private households in England. The sample size was 11 391 people at the first wave in 2002.^17^ We included 9213 participants aged 50–75 at the first wave with valid data on self-rated health and chronic diseases.

The Finnish data came from five waves of the Finnish Public Sector study (FPS). The FPS, established in 1997/1998, comprises all employees with ≥6 month job contract in any year from 1991/2000 to 2005 in 10 towns and 5 hospital districts in Finland. Survey data were collected at 4-year intervals on 103 866 cohort members, employed in the participating organizations during the survey years 1997/1998, 2000/2002, 2004, 2008 and/or 2012. Follow-up surveys for respondents who had retired or left the organizations were conducted in 2005, 2009 and 2013. Data from 42 978 people aged 50–75 at the first wave with valid health measures were analyzed.

The French data were taken from the GAZEL Cohort Study, set up in 1989 among Électricité de France-Gaz de France workers, the French national utility company, with annual waves of data collection up to 2014. At inception in 1989, the GAZEL Cohort Study included 20 625 volunteers (15 011 men and 5614 women) aged 35–50 years.^18^ Data from 18 263 people from the first wave they were aged 50 plus and had health data were analyzed.

The data for Sweden were from five waves of the Swedish Longitudinal Occupational Survey of Health (SLOSH).^19^ The SLOSH cohort is drawn from the Swedish Work Environment Survey (SWES), a cross-sectional, biennial survey of a random stratified sample of employed people aged 16–64 years who responded to the Labour Force Survey in the same year. The first wave of SLOSH in 2006 was a follow-up of respondents to the 2003 SWES. At wave 2 in 2008, new respondents to the 2005 SWES were added, giving an overall sample of 18 915. This sample was re-surveyed in 2010, 2012 and 2014. Data from 8186 people aged 50–75 were analyzed.

### Outcome measures

We defined two outcomes: (i) healthy life expectancy using self-rated health and (ii) chronic disease-free life expectancy.

*Self-rated health:* All participants were asked about their health status at each wave. Responses were categorized into good and poor health as follows. GAZEL used an eight point scale, where the participant indicated whether her/his health was very good (8) to very bad (1). In line with previous research,^20^ the four top response options were coded as good health. In ELSA, FPS and SLOSH the question used a five point response format, ranging from excellent/very good to very poor. For FPS and SLOSH, the top two response options were grouped and categorized as ‘good health’, and in ELSA, the top three response options were categorized as ‘good health’. The question wordings for each cohort are given in the online Supplementary appendix.

*Chronic diseases:* Presence of the following chronic diseases was ascertained for each cohort by asking ‘has a doctor ever told you that you have …’: (i) heart disease (heart attack, coronary heart disease, angina, congestive heart failure or other heart problems), (ii) stroke (stroke or transient ischaemic attack), (iii) chronic lung disease (chronic bronchitis or emphysema or asthma), (iv) cancer (cancer or a malignant tumour of any kind except skin cancer) and (v) diabetes (diabetes or high blood sugar). Individuals were defined as having a chronic disease if they reported one or more of these conditions. The presence of chronic disease at baseline (first observation included in analysis) included any chronic diseases reported before the age of 50 from available information on respondents. The data for Sweden on chronic disease came from 2008 to 2014 waves as the 2006 wave did not collect information on all chronic conditions.

*Mortality* was ascertained from linked register data with follow-up censored on 31 December of the year in which data collection last took place for each cohort.

### Occupational position

Occupational position was coded into three groups according to the national occupational classification: higher, intermediate and lower occupational positions. In ELSA and SLOSH, occupational position was based on self-reported job title; in FPS and GAZEL occupational position was obtained from the employers’ records.

### Statistical analyses

Characteristics of the participating cohorts are presented at the first observation point, which refers to the date each participant is first included in this analysis.

Multistate life table models were used to estimate healthy life expectancy and chronic disease-free life expectancy between the ages of 50 and 75 (in total 26 years). For both measures, three health states were defined: healthy, unhealthy and dead. For self-rated health, there were four possible transitions between these health states, namely: healthy to unhealthy (onset), unhealthy to healthy (recovery), healthy to dead, unhealthy to dead. For chronic disease, there were only three possible transitions as, by definition, recovery was not possible. The analysis included all study participants with at least one measure of health when they were aged 50 years or older. For each study, age-specific transition probabilities by sex and occupational position were estimated from multinomial logistic models with age (in years), sex and occupational position as covariates. Partial life expectancy, healthy life expectancy and chronic disease-free life expectancy from ages 50 to 75 were then calculated from these estimated transition probabilities using a stochastic (micro-simulation) approach.^21^ For each study, individual trajectories for a simulated cohort of 100 000 persons were generated with distributions of covariates at the starting point based on the observed study-specific prevalence by five year age-group, sex and occupational position. Health expectancies from age 50 to 75 were calculated as the average from these trajectories for each occupational position and sex. Computation of standard errors and 95% confidence intervals (from 2.5th and 97.5th percentiles) for these multistate life table estimates was performed using a bootstrap method with 500 replicates for the whole analysis process (multinomial analysis and simulation steps). Analyses were conducted in SAS 9.2 using the Stochastic Population Analysis of Complex Events programme (http://www.cdc.gov/nchs/data_access/space.htm).^21^

### Ethics approval

In all cohorts, participants gave their informed consent to take part. Ethical approval was given in each of the countries from relevant ethical committees/boards.

## Results

At the first observation point when participants were included in the analysis, the prevalence of poor self-rated health ranged from 20% in France to 36% in Finland. Prevalence of self-reported chronic diseases ranged from 21% in France to 33% in England (table 1).

**Table 1:** Prevalence (%) of sociodemographic and health characteristics at the first observation point^a^

	ELSA	FPS	GAZEL	SLOSH
Sample size	9213	42 978	18 263	8186
Sex (%)				
Male	46.4	19.9	73.8	45.7
Female	53.6	80.1	26.2	54.3
Age-group (%)				
50–54	21.9	70.5	94.5	41.8
55–59	24.2	24.9	3.9	24.2
60–64	18.7	4.5	1.3	23.5
65–69	18.9	0.1	0.3	10.2
70–74	16.3	–	0.1	0.3
Socioeconomic position (%)				
High grade	30.0	29.9	14.1	19.3
Middle grade	23.5	49.0	57.8	44.7
Low grade	46.5	21.2	28.2	36.0
Self-rated health (%)				
Good	75.0	63.4	79.9	77.9
Poor	25.0	36.0	20.1	22.1
Chronic health conditions^b^				
No	67.1	74.1	79.5	80.7
Yes	32.9	25.9	20.5	19.3

a The first observation point refers to the date each participant is for the first time included in the dataset.

b Presence of chronic health conditions includes illness reported at or before the first observation point.

For all cohorts, the risk of mortality or poor self-reported health from a state of good self-reported health was higher for people in the lowest occupational group. Correspondingly, the likelihood of recovery from poor health was lower for people in the lowest occupational group (Supplementary table S1). Risk of mortality from a poor health state also showed a similar gradient by occupational position.

### Socioeconomic differences in healthy life expectancy

There were occupational position differences for partial life expectancy and healthy life expectancy at age 50 in all cohorts such that people in the lower occupational position could expect to live fewer total years to age 75 and to spend fewer of these years in good health (table 2). These differences were more marked for healthy life expectancy than partial life expectancy for all cohorts. For example, women in England with a high occupational position could expect to spend 83% of their life from 50 to 75 in good health whereas the corresponding figure for English women in a lower occupational position was 68%. Our estimates of occupational position differences in healthy years of life lost between the ages of 50–75 ranged from 2.1 years for women in the Swedish cohort to 6.8 years for men in the Finnish cohort.

**Table 2:** Partial life expectancy, healthy life expectancy and proportion of life spent in good self-rated health between the ages of 50 and 75 by socioeconomic position

	Partial life expectancy between the ages of 50 and 75						
	Life expectancy	95% CI	Healthy life expectancy	95% CI	Unhealthy life expectancy	95% CI	% spent in good health
Men							
ELSA							
High grade	24.1	24.0, 24.4	19.8	19.4, 20.3	4.3	4.0, 4.7	82.2
Middle grade	23.7	23.4, 24.0	18.2	17.6, 18.9	5.5	4.9, 5.9	76.8
Low grade	22.9	22.6, 23.3	15.5	14.9, 16.1	7.4	7.0, 7.9	67.7
*High−low*	*1.2*		*4.3*		*−3.1*		*14.5*
FPS							
High grade	24.5	24.4, 24.7	18.5	18.2, 19.0	6.0	5.6, 6.3	75.4
Middle grade	24.1	23.9, 24.4	15.4	15.1, 16.0	8.7	8.2, 9.1	63.8
Low grade	23.4	23.2, 23.9	11.7	11.3, 12.3	11.7	11.2, 12.2	49.9
*High−low*	*1.1*		*6.8*		*−5.7*		*25.5*
GAZEL							
High grade	24.8	24.6, 25.0	21.6	21.3, 21.8	3.2	3.0, 3.4	87.1
Middle grade	24.3	24.2, 24.4	20.5	20.3, 20.7	3.9	3.7, 4.0	84.2
Low grade	23.8	23.6, 24.0	18.8	18.6, 19.1	5.3	5.0, 5.6	79.1
*High−low*	*1.0*		*2.8*		*−2.1*		*8.0*
SLOSH							
High grade	25.6	25.4, 25.9	20.4	19.8, 21.1	5.1	4.5, 5.8	79.9
Middle grade	25.2	24.9, 25.5	19.7	19.2, 20.4	5.5	4.9, 5.9	78.2
Low grade	25.3	24.9, 25.5	18.1	17.4, 18.6	7.2	6.6, 7.8	71.6
*High−low*	*0.3*		*2.3*		*−2.1*		*8.3*
Women							
ELSA							
High grade	24.8	24.6, 25.0	20.6	20.0, 21.0	4.2	3.9, 4.8	83.0
Middle grade	24.5	24.2, 24.7	19.1	18.6, 19.6	5.4	4.9, 5.9	78.1
Low grade	23.9	23.6, 24.2	16.1	15.5, 16.7	7.8	7.3, 8.3	67.5
*High−low*	*0.9*		*4.5*		*−3.6*		*15.5*
FPS							
High grade	25.1	25.0, 25.3	18.9	18.7, 19.2	6.2	6.0, 6.5	75.3
Middle grade	24.9	24.8, 25.0	16.3	16.1, 16.6	8.6	8.4, 8.8	65.5
Low grade	24.6	24.4, 24.8	12.4	12.1, 12.8	12.2	11.8, 12.5	50.5
*High−low*	*0.5*		*6.5*		*−6.0*		*24.8*
GAZEL							
High grade	25.3	25.1, 25.5	21.6	21.2, 21.9	3.7	3.4, 4.1	85.4
Middle grade	25.0	24.8, 25.2	20.5	19.9, 20.4	4.5	4.3, 4.7	82.0
Low grade	24.7	24.5, 24.9	18.9	18.0, 18.8	5.7	5.6, 6.0	76.7
*High−low*	*0.6*		*2.7*		*−2.0*		*8.7*
SLOSH							
High grade	25.7	25.5, 25.9	21.3	20.7, 21.9	4.4	3.9, 5.0	82.8
Middle grade	25.4	25.3, 25.6	20.8	20.3, 21.1	4.7	4.4, 5.0	81.7
Low grade	25.5	25.1, 25.8	19.2	18.6, 19.6	6.3	5.8, 6.8	75.3
*High−low*	*0.2*		*2.1*		*−1.9*		*7.5*

### Socioeconomic differences in disease-free life expectancy

Results from the multistate models for chronic diseases (Supplementary table S2) showed socioeconomic differences in risk of transition to chronic disease or mortality from a disease-free state as well as higher risk of mortality from having a chronic disease. For each cohort, our estimates of chronic disease-free life expectancy (table 3) showed that, compared to those in a higher occupational position, men and women in the lower occupational position could expect to live fewer years without chronic diseases between ages 50 and 75. For example, women in England in the higher occupational grade could expect to spend 61% of their life from 50 to 75 without chronic disease. The corresponding figure for women in the lower occupational grade in England was 56%. Our estimates of occupational position differences in chronic disease-free years of life lost between the ages of 50–75 ranged from 0.5 years for women in the French cohort to 2.3 years for men in the Finnish cohort.

**Table 3:** Partial life expectancy, chronic disease-free life expectancy and proportion of life spent without chronic disease between the ages of 50 and 75 by socioeconomic position

	Partial life expectancy between the ages of 50 and 75						
	Life expectancy^a^	95% CI	Chronic disease-free life expectancy	95% CI	Life expectancy with chronic diseases	95% CI	% spent without chronic disease
Men							
ELSA							
High grade	24.1	23.9, 24.3	13.8	12.7, 14.9	10.3	9.3, 11.3	57.4
Middle grade	23.6	23.3, 24.0	13.2	11.6, 14.4	10.4	9.4, 11.8	56.1
Low grade	23.1	22.8, 23.4	12.8	11.5, 13.8	10.3	9.4, 11.5	55.2
*High−low*	*1.0*		*1.0*		*0.0*		*2.2*
FPS							
High grade	24.4	24.2, 24.7	13.7	12.9, 13.9	10.8	10.5, 11.6	55.9
Middle grade	24.1	23.6, 24.3	12.4	11.7, 12.9	11.7	11.1, 12.3	51.3
Low grade	23.5	23.2, 23.8	11.4	11.0, 11.9	12.0	11.4, 12.6	48.7
*High−low*	*0.9*		*2.3*		*−1.2*		*7.2*
GAZEL							
High grade	24.8	24.6, 25.0	15.5	15.1, 16.1	9.3	8.8, 9.7	62.5
Middle grade	24.3	24.2, 24.4	14.9	14.6, 15.2	9.4	9.1, 9.7	61.3
Low grade	23.8	23.6, 24.0	13.9	13.6, 14.4	9.9	9.4, 10.2	58.4
*High−low*	*1.0*		*1.6*		*−0.6*		*4.1*
SLOSH							
High grade	25.6	25.4, 25.9	15.0	13.6, 16.5	10.7	9.1, 12.1	58.4
Middle grade	25.1	24.7, 25.4	14.5	13.3, 15.6	10.6	9.6, 11.8	57.8
Low grade	25.1	24.7, 25.4	13.6	12.6, 14.5	11.6	10.6, 12.5	53.9
*High−low*	*0.5*		*1.4*		*−0.9*		*4.5*
Women							
ELSA							
High grade	24.8	24.6, 24.9	15.2	13.8, 16.5	9.6	8.4, 10.9	61.3
Middle grade	24.5	24.3, 24.7	15.2	13.6, 16.4	9.2	8.1, 10.8	62.2
Low grade	24.0	23.8, 24.3	13.4	12.2, 14.6	10.6	9.4, 11.9^16^	55.9
*High−low*	*0.8*		*1.8*		*−1.0*		*5.4*
FPS							
High grade	25.1	25.0, 25.2	13.3	12.9, 13.7	11.9	11.5, 12.2	52.8
Middle grade	24.9	24.8, 25.0	13.5	13.1, 13.6	11.4	11.3, 11.8	54.0
Low grade	24.5	24.4, 24.8	12.6	12.2, 13.1	11.9	11.5, 12.5	51.5
*High−low*	*0.6*		*0.7*		*0.0*		*1.3*
GAZEL							
High grade	25.3	25.1, 25.5	15.7	14.8, 16.9	9.7	8.3, 10.5	61.9
Middle grade	25.0	24.8, 25.1	15.6	15.2, 16.0	9.4	9.0, 9.8	62.4
Low grade	24.7	24.5, 24.9	15.2	14.8, 15.7	9.5	9.0, 9.9	61.6
*High−low*	*0.6*		*0.5*		*0.2*		*0.3*
SLOSH							
High grade	25.8	25.6, 26.0	16.7	15.5, 18.2	9.1	7.5, 10.3	64.9
Middle grade	25.4	25.1, 25.6	17.0	16.0, 17.8	8.4	7.5, 9.2	66.9
Low grade	25.4	25.0, 25.6	15.3	14.3, 16.5	10.1	8.9, 11.0	60.3
*High−low*	*0.4*		*1.4*		*−1.0*		*4.6*

a Due to the micro-simulation approach, the partial LE presented in this column may differ slightly from those in table 2.

### Sensitivity analyses

We conducted several sensitivity analyses. Estimates of healthy and chronic disease-free life expectancy were very similar for occupational socioeconomic position recorded at either age 35 or at age 50. We investigated the potential impact of frequency of follow-up on our results by repeating analyses for GAZEL with data from every fourth wave rather than yearly intervals and found very similar results. As chronic conditions were considered to be non-reversible, the estimates of prevalence of chronic disease by age 50 tended to be higher for participants with more waves of data before age 50. Therefore, we repeated analyses excluding information on chronic conditions collected before the age of 50. As expected, this led to higher estimates of years lived without chronic disease but differences by socioeconomic position remained similar for each cohort. Finally, we obtained a similar pattern of results from analyses including information on chronic diseases from both register and self-report data for the Finnish and Swedish cohorts.

## Discussion

This cross-national multi-cohort study found socioeconomic inequalities in both partial life expectancy and healthy life expectancy such that socioeconomic differences in years of healthy life lost are greater than differences in years of life lost between the ages of 50 and 75. Compared to people in higher occupational positions, those in lower occupational positions had a lower expectancy of life years until age 75 and could expect to live fewer of these years in good health. This pattern was consistent across the four countries and observed for both men and women. There were also socioeconomic inequalities in chronic disease-free life expectancy, although estimates of average years lived without diseases were lower than for average years lived in good perceived health.

Our findings are in line with previous studies that have used education as a measure of socioeconomic inequality that show that those with the least level of education have the lowest health expectancies.^7–12^ In our study, men and women in the lowest compared to the highest occupational position could expect 2.1–6.8 fewer years in good health and 0.5–2.3 fewer years lived without chronic disease between the ages of 50 and 75.

To our knowledge, few previous studies have reported differences in health expectancies by occupational social position. These studies also found socioeconomic inequalities in health expectancies but there were some differences when comparing inequalities for men and women. A previous study of socioeconomic inequalities in disability-free life expectancy in the UK found greater social class inequalities amongst women compared to men.^15^ This is in line with our results for chronic disease where the difference in chronic disease-free life expectancy between the low and high occupational class was 1.8 years for English women and 1.0 years for English men but not consistent with our results for self-rated health where we found similar occupational class inequalities in men and women. As with the current study, an earlier study in France found similar social class differences in healthy life expectancy for men and women.^14^ However, social class differences in chronic disease-free life expectancy at age 50 were wider for French women (4.4 years) than for French men (3.0 years) in the earlier study whereas we found slightly wider social class differences for French men (1.6 years) than for French women (0.5 years). This may reflect methodological differences as the previous study used information from a national survey and their measure of chronic disease was based on a single question.

Estimates of absolute socioeconomic differences in healthy and chronic disease-free life expectancy varied across the four cohorts and were most marked in the Finnish cohort, particularly for self-rated health. Although there have been two previous cross-national studies of socioeconomic inequalities in health expectancies, these investigated educational inequalities in disability-free life expectancy.^8^^,^^9^ As stated by Pongiglione et al. in their systematic literature review of inequalities in health expectancies, estimates of years of healthy life lost tend to vary according to the definition of health making it difficult to compare results from different studies.^7^ Our finding that estimates of average years lived without chronic disease were lower than for average years lived in good health was consistent with findings from studies with multiple health indicators included in the review by Pongligione et al.^7^

Strengths of our study include the use of data from prospective cohorts from four different countries, with several measurements of health over time, long follow-up, and high quality harmonised data. Our multistate life table method of estimating healthy life expectancy and chronic disease-free life expectancy using longitudinal data provides internally consistent results for each cohort.

However, there were some limitations. Not all cohorts were nationally representative. Whereas ELSA is a national survey, the others were occupational cohorts that were either designed to be representative of all employees (SLOSH) or specific employment sectors (FPS and GAZEL). Participants in longitudinal studies tend to be healthier than the general population and more disadvantaged groups tend to be underrepresented, and additionally there were socioeconomic differences in attrition rates.^22–24^ However, past research from our studies have shown similar socioeconomic differentials in health to those seen nationally implying that our results are generalizable to a wider population.^25^ The cohorts did not have entirely consistent definitions of self-rated health and this could contribute to some of the cohort differences in absolute healthy life expectancy. However, relative differences by socioeconomic group were consistent across the cohorts. We used five chronic diseases, namely heart disease, stroke, chronic lung disease, cancer and diabetes, to estimate chronic disease-free life expectancy. Although musculoskeletal disorders were measured in each cohort, it was not possible to develop a harmonized measure of musculoskeletal disorders across the cohorts. As these disorders are very common at older ages and related to poorer functioning and quality-of-life, future studies of chronic disease-free life expectancy should include musculoskeletal disorders.

There were differences in frequency of follow-up intervals between studies ranging from annual to four yearly waves of data collection. To assess the possible impact of this, we repeated analyses for the GAZEL cohort using data from every fourth wave and obtained similar results. Our study provides estimates of healthy life expectancy and chronic disease-free life expectancy from age 50 to 75 so future studies are needed to investigate socioeconomic differences in years of life spent in good health and without chronic disease at older ages. Finally, we were not able to analyze changes over time in health expectancies by socioeconomic position. Results from a study comparing health expectancies in 1991 and 2011 in England showed a relative compression of morbidity for self-perceived health, with an increase in the proportion of life spent in good health.^26^ Future research is needed to investigate socioeconomic differences in changes in health expectancies over time.

## Conclusions

A better understanding of the future health of older people is of crucial policy importance as it affects public expenditure on income, health and long-term care needs of ageing populations.^27^ Furthermore, healthy and disease-free life expectancy is also important for work participation, given the actions by most European governments to extend working lives.^28^ Our study highlights the importance of reducing social class differences in health expectancies as part of efforts to achieve that target.

## Funding

This study was supported by the Swedish Research Council for Health for Working Life and Welfare (2007-1762, 2009-1758 and 2012-1661); the Academy of Finland (264944); and the UK Economic and Social Research Council [ES/K01336X/1] under the ERA-AGE2 initiative. J.H. was partly supported by the ESRC (ES/L0028921/1). S.S. is supported by the Academy of Finland (286294 and 294154). M.K. was supported by the UK Medical Research Council (MR/K013351/1), the Economic and Social Research Council and NordForsk, the Nordic Programme on Health and Welfare.

## Disclaimer

The sponsors had no role in the design and conduct of the study; collection, management, analysis and interpretation of the data; and preparation, review or approval of this manuscript.

*Conflicts of interest:* None declared.

**Key points**

- Few studies have examined socioeconomic differences in health expectancies and most of these have used education as a measure of socioeconomic position.
- Occupational socioeconomic position may be a more relevant measure, particularly in the context of extending working lives.
- There were inequalities in healthy life expectancy and chronic disease-free life expectancy between ages 50 and 75 according to occupational position.
- Men and women in low occupational positions had a lower expectancy of life years until age 75 and could expect to live fewer of these years in good health or without chronic disease.
- Reducing socioeconomic differences in health expectancies should be part of policies aimed at extending the working lives of people aged 50 and over.

## Supplementary Material

Supplementary Material
