# Supplementary material for: Socioeconomic differences in healthy and disease-free life expectancy between ages 50 and 75: a multi-cohort study
Source: Eur J Public Health. 2018 Oct 10;29(2):267–72. doi: 10.1093/eurpub/cky215 (PMC6426044; doi:10.1093/eurpub/cky215)
Supplement: Supplementary Material [file cky215_supplementary_material.docx]

**Supplementary material**

**Title: Socioeconomic differences in healthy and disease-free life expectancy between ages 50 and 75: a multicohort study**

**Authors**: Jenny Head, Holendro Singh Chungkham, Martin Hyde, Paola Zaninotto, Kristina Alexanderson, Sari Stenholm, Paula Salo, Mika Kivimäki, Marcel Goldberg, Marie Zins, Jussi Vahtera, Hugo Westerlund*.*

**Corresponding author:**

Jenny Head,

Department of Epidemiology and Public Health,

University College London,

1 - 19 Torrington Place London,

WC1E 6BT, UK

Email: j.head@ucl.ac.uk,

**Appendix: Wording of self-rated health questions and response options in each cohort**

*English Longitudinal Study of Ageing (ELSA)*

Would you say your health is...?

1 ‘excellent’,

2 ‘very good’,

3 ‘good’,

4 ‘fair’,

5 or, ‘poor’?

*Finnish Public Sector Study (FPS)*

How is your health?

1 'good'

2 'fairly good'

3 'average'

4 'fairly poor'

5 'poor'

*GAZEL*

“How would you judge the state of your general health?”

Responses on 8-point Likert scale from 1 'very good' to 8 'very poor'

*Swedish Longitudinal Occupational Survey of Health (SLOSH)*

How would you rate your general state of health?

1 'very good'

2 'quite good'

3 'neither good nor bad'

4 'quite poor'

5 'very poor'

**Table S1. Country specific odds ratios** ^a^ **for self-reported health transitions from multinomial logistic models**

|  | Healthy to unhealthy | Unhealthy to healthy | Healthy to  death | Unhealthy to  death |
| --- | --- | --- | --- | --- |
|  | OR (95% CI) | OR (95% CI) | OR (95% CI) | OR (95% CI) |
| England |  |  |  |  |
| High grade | 1.00 | 1.00 | 1.00 | 1.00 |
| Middle grade | 1.26 (1.13 to 1.40) | 0.88 (0.77 to 1.00) | 1.19 (0.98 to 1.45) | 1.14 (0.94 to 1.39) |
| Low grade | 1.75 (1.60 to 1.92) | 0.67 (0.60 to 0.74) | 1.64 (1.39 to 1.94) | 1.18 (1.00 to 1.38) |
| Finland |  |  |  |  |
| High grade | 1.00 | 1.00 | 1.00 | 1.00 |
| Middle grade | 1.46 (1.36 to 1.58) | 0.76 (0.71 to 0.82) | 1.25 (0.94 to 1.65) | 1.34 (1.01 to 1.77) |
| Low grade | 2.21 (2.03 to 2.40) | 0.56 (0.52 to 0.62) | 1.94 (1.44 to 2.61) | 1.29 (0.97 to 1.72) |
| France |  |  |  |  |
| High grade | 1.00 | 1.00 | 1.00 | 1.00 |
| Middle grade | 1.23 (1.17 to 1.29) | 0.93 (0.87 to 0.99) | 1.47 (1.15 to 1.89) | 1.19 (0.93 to 1.53) |
| Low grade | 1.52 (1.45 to 1.61) | 0.74 (0.69 to 0.79) | 2.05 (1.57 to 2.68) | 1.11 (0.85 to 1.44) |
| Sweden |  |  |  |  |
| High grade | 1.00 | 1.00 | 1.00 | 1.00 |
| Middle grade | 1.02 (0.88 to 1.18) | 0.92 (0.78 to 1.07) | 2.01 (0.87 to 4.64) | 1.98 (0.56 to 7.00) |
| Low grade | 1.31 (1.13 to 1.51) | 0.78 (0.67 to 0.92) | 1.19 (0.47 to 3.03) | 2.50 (0.73 to 8.51) |

^a^ adjusted for age and sex

**Table S2. Country specific odds ratios** ^a^ **for chronic disease transitions from multinomial logistic models**

|  | Disease-free to disease | Disease-free to death | Disease to  death |
| --- | --- | --- | --- |
|  | OR (95% CI) | OR (95% CI) | OR (95% CI) |
| England |  |  |  |
| High grade | 1.00 | 1.00 | 1.00 |
| Middle grade | 0.94 (0.82 to 1.07) | 1.22 (0.92 to 1.62) | 1.20 (1.03 to 1.41) |
| Low grade | 1.06 (0.94 to 1.19) | 1.58 (1.24 to 2.01) | 1.50 (1.31 to 1.71) |
| Finland |  |  |  |
| High grade | 1.00 | 1.00 | 1.00 |
| Middle grade | 1.08 (1.01 to 1.16) | 1.57 (1.20 to 2.06) | 1.28 (0.96 to 1.71) |
| Low grade | 1.35 (1.25 to 1.47) | 2.12 (1.59 to 2.81) | 1.57 (1.17 to 2.10) |
| France |  |  |  |
| High grade | 1.00 | 1.00 | 1.00 |
| Middle grade | 1.12 (1.04 to 1.21) | 1.42 (1.03 to 1.97) | 1.46 (1.19 to 1.80) |
| Low grade | 1.10 (1.01 to 1.20) | 1.84 (1.28 to 2.65) | 1.91 (1.54 to 2.38) |
| Sweden |  |  |  |
| High grade | 1.00 | 1.00 | 1.00 |
| Middle grade | 1.12 (0.98 to 1.30) | 1.95 (0.64 to 5.99) | 2.13 (0.81 to 5.63) |
| Low grade | 1.35 (1.16 to 1.57) | 2.07 (0.65 to 6.60) | 1.95 (0.72 to 5.27) |

^a^ adjusted for age and sex
